# Effect of Heat Treatment on the Microstructure and Property of Metastable β Titanium Alloy

**DOI:** 10.3390/ma17246294

**Published:** 2024-12-23

**Authors:** Jiafeng Tang, Hengjun Luo, Biliu Wu, Wenhao Liu, Yu Rong, Danyang Chen, Yulin Qin, Ning Zhang, Fang Hao, Hao Deng, Longqing Chen, Jun Zhu, Ming Yin

**Affiliations:** 1 College of Physics, Sichuan University, Chengdu 610065, China; tangjiafeng1234@163.com (J.T.); sculiuwenhao@163.com (W.L.); qinyl163@163.com (Y.Q.); zhangning101230@163.com (N.Z.); zhujun01@163.com (J.Z.); 2 China National Erzhong Group Deyang Wanhang Die Forging Co., Ltd., Deyang 618099, China; luohengjun2022@163.com; 3 School of Materials Engineering, Sichuan Polytechnic University, Deyang 618000, China; bellawu1995@163.com; 4 State Grid Shanxi Electric Power Company, Taiyuan 030000, China; 15333663957@163.com (Y.R.); chendanyangsj@163.com (D.C.); 5 Western Superconducting Technologies Co., Ltd., Xi’an 710018, China; haofang85@163.com; 6 Key Laboratory of Radiation Physics and Technology of Ministry of Education, Institute of Nuclear Science and Technology, Sichuan University, Chengdu 610064, China; 7 School of Mechanical Engineering, Sichuan University, Chengdu 610064, China; mingyin@scu.edu.cn

**Keywords:** near β titanium alloy, solution treatment, aging treatment, α precipitation, mechanical properties

## Abstract

TB18 is a newly developed high-strength metastable β-titanium alloy, commonly used in aerospace structural materials, which demands high mechanical performance. By altering the alloy’s microstructure, heat treatment can affect its mechanical characteristics. The alloy was solution treated for one to four hours at 870 °C in order to examine the impact of solution treatment duration. Using X-ray diffraction (XRD) and scanning electron microscopy (SEM), the effects of solution treatment time on the β-phase grain size and its effect on stress distribution during tensile testing were examined. The findings showed that stress concentration during the tensile process was successfully decreased by refining the β-phase grain size. Sample solutions treated for two hours at 870 °C were then aged at various temperatures (510 °C, 520 °C, 530 °C, and 540 °C) to examine the impact of aging temperature. While the mass proportion of the α-phase first climbed and subsequently declined, reaching its maximum at 530 °C, the size of the α-phase increased monotonically as the aging temperature increased. The varies of mass fraction is associated with how the aging temperature affects α-phase nucleation. Tensile studies on TB18 alloy aged at various temperatures showed that while the alloy’s ductility reduced, its strength increased as the aging temperature rose. The Hall-Petch relationship explains this tendency.

## 1. Introduction

Because of their exceptional corrosion resistance, resistance to deep hardening, and high specific strength, metastable β titanium alloys are used extensively in the orthopedic implant and aerospace sectors [1,2,3,4,5,6]. The near-β titanium alloy TB18 (Ti-5.3Cr-4.9Mo-4.9V-4.3Al-0.9Nb-0.3Fe) is enhanced with β-stabilizing elements like Mo and Cr. Because of its exceptional strength and ductility, it is frequently used in aeronautical structural elements like landing gears and wings [7,8,9,10].

Nevertheless, their remarkable strength frequently results in decreased ductility [11]. Heat treatment can be used to modify the microstructure of metastable β alloys to achieve the ideal balance between strength and ductility. This procedure usually entails aging and solution treatment [2,12,13]. While aging treatment can effectively enhance the disintegration of β phase to create fine α phase, solution treatment can influence the stability of β phase and β grain size [8,14,15]. The alloy’s strength and ductility are greatly impacted by the α phase [16,17], whereas its ductility is impacted by the β phase [11,18,19]. Therefore, achieving the appropriate mechanical properties in β titanium alloys requires careful management of microstructural changes after heat treatment.

The characteristics of metastable β alloys and the subsequent precipitation of the α phase are greatly impacted by changes in their microstructure that occur during solution treatment in the β region [8,14,20]. For instance, Wang et al. found that a higher solution temperature results in a bigger β phase size and less ductility [11]. According to Fan et al.’s research, extended solution times also cause β grain size to grow and ductility to decrease [21]. The microstructure of the α phase is changed by aging at varying temperatures, which affects its ductility and strength [12]. Higher aging temperatures resulted in a thicker secondary α layer, which decreased alloy hardness, according to a recent study [22]. Furthermore, Chen et al. observed that while increasing the aging temperature initially increased the alloy’s strength because the secondary α phase did not precipitate sufficiently, doing so resulted in a decrease in strength because of secondary α phase coarsening [23]. Therefore, choosing the right aging conditions is essential to guarantee full α phase precipitation. The mechanical properties of the alloy are greatly impacted by the solution and aging conditions chosen, which highlights the significance of examining their impact on metastable β alloys. Further research is needed, since TB18 alloy has amazing mechanical properties that can be well-regulated through solution treatment and age treatment.

In this study, The TB18 alloy was solution-treated for one to four hours at 870 °C. Furthermore, samples that were solution-treated for two hours at 870 °C underwent age treatments at 510 °C, 520 °C, 530 °C, and 540 °C. Using a variety of characterization techniques, such as optical microscopy (OM), X-ray diffraction (XRD), scanning electron microscopy (SEM), electron backscatter diffraction (EBSD), and transmission electron microscopy (TEM), the goal of this study is to examine how heat treatment affects the alloy’s microstructure. The study evaluates how these microstructural variations impact the alloy’s mechanical characteristics and investigates the underlying mechanisms behind the microstructure changes brought on by the heat treatment process. Lastly, the connection between the alloy’s mechanical performance and microstructure is established. The results of this study will offer a useful reference for further investigations aimed at enhancing the functionality of TB18 alloy for cutting-edge uses.

## 2. Experiments and Methods

### 2.1. Material

**Figure 1:**
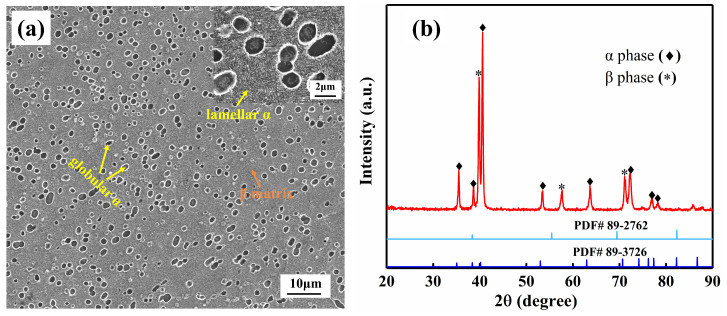
SEM micrographs and XRD pattern of received TB18 alloy. (**a**) SEM micrographs of TB18 alloy; (**b**) XRD image of received TB18 alloy. The yellow arrows in the Figure 1 (**a**) represent the α phase, and the orange arrows represent the β phase.

Wan Hang Die Forging provided the TB18 alloy used in this investigation. The embryo is a rod-shaped forging heated to 765 °C to 770 °C. The alloy’s chemical composition was identified as Ti-5.3Cr-4.9Mo-4.9V-4.3Al-0.9Nb-0.3Fe. Through metallographic examination, the alloy’s β phase transition temperature (Tβ) was discovered to be around 800 °C. The forging alloy is formed of both β and α phases, as shown in Figure 1b. The alloy’s microstructure is shown in Figure 1a, where the fine lamellar α and tiny globular α are evenly dispersed throughout the β matrix.

### 2.2. Heat Treatment (HT) Scheme

An air box furnace was used for the solution and aging processes. Table 1 describes the heat treatment plans used in this investigation. The HT 1 scheme was created with the intention of examining how solution time affects the β grain and the mechanical characteristics of TB18 alloy. In HT 1, the sample was heated at a rate of 10 °C per minute to a temperature of 870 °C (Tβ + 70 °C), above the phase transition temperature. Various solution times of one, two, three, and four hours were used. All samples underwent air cooling (AC) following heat treatment at HT1. These samples treated with the solution were then aged for four hours at 530 °C. The aim of the HT 2 scheme was to examine how the aging temperature affected the α phase’s precipitation and the subsequent mechanical characteristics of the TB18 alloy. Every sample in the HT 2 scheme underwent a two-hour solution treatment at 870 °C. After four hours of aging at various temperatures (510 °C, 520 °C, 530 °C, and 540 °C), the samples were cooled by AC.

### 2.3. Microstructural Characterization

To explore the microstructure of the alloys, various characterization techniques were employed: optical microscope (OM, DMi8 M/C/A, Ernst Leitz Company, Wetzlar, Germany); scanning electron microscope (SEM, JSM-IT500HR, Tokyo, Japan); X-ray diffraction (XRD, X’Pert PRO instrument, Almelo, The Netherlands); electron backscattered diffraction (EBSD, Nordlys Nano system from Oxford Instruments in Oxford, UK), and; transmission electron microscope (TEM, F20 apparatus from FEI, Hillsboro, OR, USA).

Samples were first metallographically polished before being etched using the Kroll reagent (2 mL HF + 25 mL HNO_3_ + 50 mL H_2_O) for SEM and OM examination [24]. The SEM test was conducted at a working distance of 13.8 mm, with an acceleration voltage of 20 kV and a probe current of 10 nA. CuKa was chosen for testing after the phase constituents were discovered using XRD, and the working voltage and current were 40 mA and 40 kV, respectively. Samples for EBSD analysis underwent metallographic polishing followed by electrochemical corrosion using a solution comprising 10% perchloric acid and 90% methanol at a voltage of 20 V for 10 s. The EBSD test was conducted with an acceleration voltage of 20 kV and the step size of 3 µm. TEM thickness of 0.3 mm was prepared by cutting and then mechanically grinding on waterproof sandpaper with a thickness of 50 μm, jet polished by a double-jet polisher in an electrolyte solution comprising perchloric acid, n-butanol, and methanol (65%:35%:59%) at low temperature (−35 °C). Utilizing Image-Pro-Plus 6.0 (IPP 6.0) analysis software, quantitative measurements of β and α grain sizes were performed.

### 2.4. Property Tests

The ASTM E8/E8M-16a standard was followed in the preparation of Standard M10 cylindrical tension test specimens to evaluate the samples’ strength and elongation [7]. Uniaxial room temperature tensile tests were then carried out using an Instron 8801 machine, with a tensile rate of 0.5 mm/min. For each situation, three tensile tests were performed, and the results were averaged to improve accuracy.

## 3. Results and Discussion

### 3.1. Effect of Heat Treatment on the Microstructure of TB18 Alloy

#### 3.1.1. Microstructures of Solution Treated Samples

**Figure 2:**
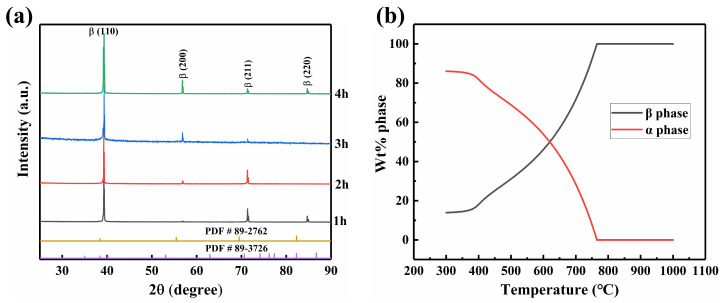
(**a**) XRD map of TB18 alloy treated with various solution times; (**b**) Temperature-mass fraction of phase diagram of TB18 titanium alloy at equilibrium conditions (By JMatPro).

Figure 2a shows the TB18 alloy’s XRD map after it was exposed to various solution times. As the solution temperature used in this study was 870 °C, the α phase completely transformed into the β phase during the solution treatment since this temperature is higher than the β phase transition point (see in Figure 2b). There is a certain difference between the simulated phase transition temperature and the experimentally tested phase transition temperature, which may be related to the uneven distribution of the alloy composition. Consequently, alloy elements did not have enough time to diffuse during the fast AC process. Consequently, the α phase’s precipitation from the β matrix is hindered. As a result, the figure only shows the diffraction peak associated with the β phase.

**Figure 3:**
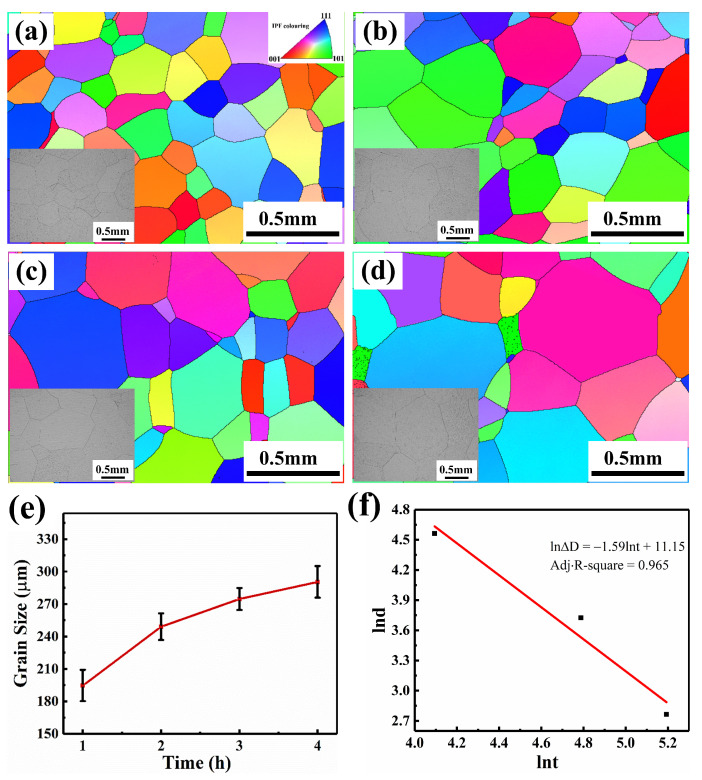
Microstructure and the size of β grain of TB18 alloy treated at 870 °C with different solution times (**a**) 1 h, (**b**) 2 h, (**c**) 3 h, (**d**) 4 h; (**e**) The change of β grain size with solution treatment time; (**f**) Simulation results of Equation (2).

The microstructure of the TB18 alloy treated with different solution times is shown in Figure 3. After the solution treatment, all of the equiaxed β grains make up the microstructures of the solution-treated samples. The average β grain sizes for different solution times of 1 h, 2 h, 3 h, and 4 h were determined to be 194 ± 14.5 μm, 249 ± 12.3 μm, 274 ± 10.2 μm, and 290 ± 14.6 μm, respectively, using the linear intercept approach [25]. The β grain size grows with longer solution times, although at a at a decelerating growth rate. In general, the following equation can be used to express grain growth [26,27].

(1) $$D_{0}=D_{t}-Kt^{n}$$

where *D_t_* is the average grain size, *D*_0_ is the initial grain size, *K* is a temperature dependent constant, and *t* is the time exponent related to solution treatment temperature and chemical composition [28]. *D* is the difference between *D*_0_ and *D_t_*, and taking the logarithm of both sides, Equation (2) is developed,

(2) lnD=lnK+nlnt

It is readily apparent there exists a linear function relationship between *lnD* and *lnt*. By substituting the experimental data from this experiment into Equation (2), where *n* equals −1.59, a negative value, it is clear that the grain size does not, in fact, change linearly with solution time. The findings also demonstrate that the TB18 alloy’s grain growth rule complies with Equation (2). Grain growth is an energy-driven process where the system spontaneously shifts toward a lower energy state, thereby reaching a more stable state. This process is associated with the migration of grain boundaries, which are typically considered to have a higher energy density. As the system tends to minimize the total Gibbs free energy, the reduction of the total grain boundary energy provides the driving force for grain growth. The grain boundary energy acts as the driving force during solution treatment at 870 °C, causing atoms to spontaneously migrate from one grain’s boundaries to the interior of nearby grains as the solution treatment time increases. Grain boundaries are reduced as a result, and grain growth occurs [26,27]. However, as grain boundaries are consumed, less boundary energy is available, which lessens the motivation for additional grain growth and, ultimately, slows the rate [21].

**Figure 4:**
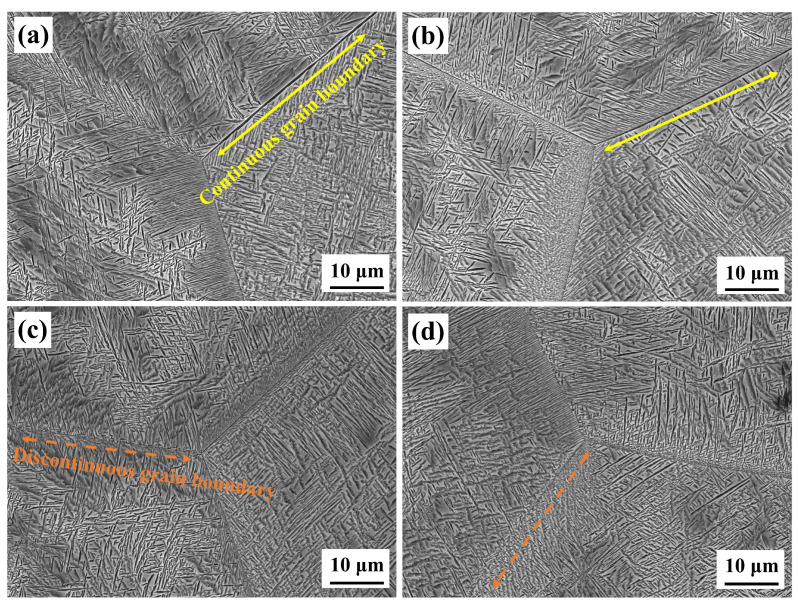
The microstructure of the samples following aging treatment with varying solution treatment times. (**a**) 1 h; (**b**) 2 h; (**c**) 3 h; (**d**) 4 h. The solid yellow arrows represent continuous grain boundaries, and the dashed orange arrows represent discontinuous grain boundaries.

The microstructure of the samples following aging treatment with varying solution treatment times is displayed in Figure 4. Every sample has a characteristic basket structure, with similar intragranular α-phase (α_s_) in morphology and size. The α-phase at the grain boundaries (α_GB_) varies significantly, though. The α_GB_ is continuous for samples treated with a solution for one or two hours, but discontinuous for samples treated with a solution for three or four hours, making it challenging to distinguish the grain boundaries.

#### 3.1.2. Precipitation of the α Phase During Aging Treatment

**Figure 5:**
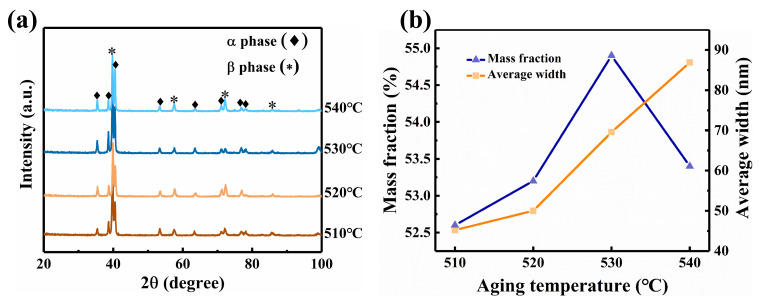
(**a**) The XRD test results of TB18 alloy with different aging temperatures treatment after the same solution treatment (870 °C/2 h); (**b**) Size and mass fraction of α_s_ for samples with different aging temperatures.

XRD analysis was performed on the alloy to examine the effect of aging temperature on phase precipitation; the findings are shown in Figure 5a. When comparing the alloy after age treatment to that before, the XRD data show the presence of the β and α phases.

**Figure 6:**
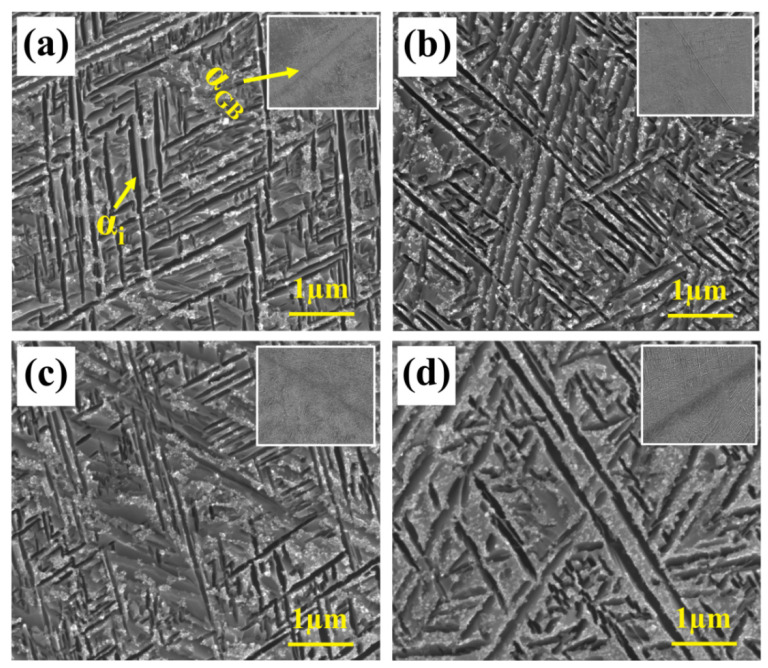
The microstructure of TB18 alloy with different aging temperatures after the same solution treatment (870 °C/2 h). (**a**) 510 °C; (**b**) 520 °C; (**c**) 530 °C; (**d**) 540 °C.

**Figure 7:**
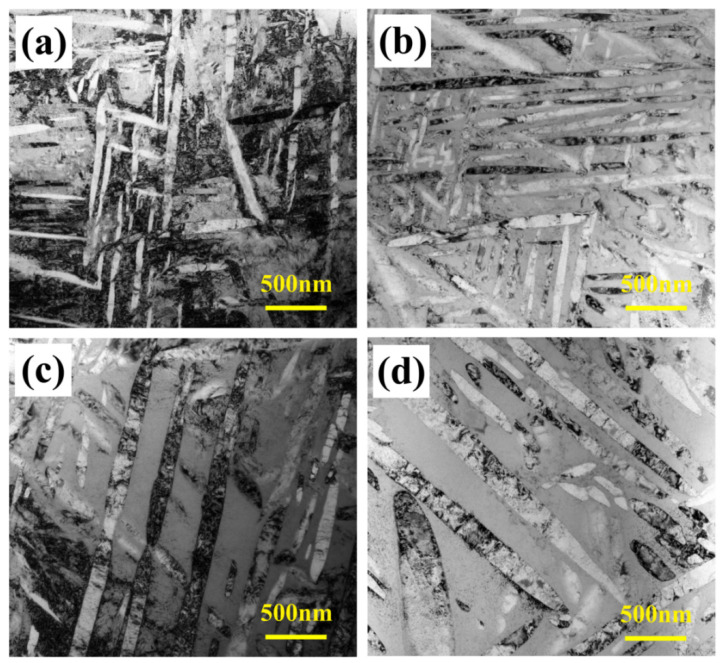
The microstructure of TB18 alloy with different aging temperatures treatment after the same solution treatment (870 °C/2 h). (**a**) 510 °C; (**b**) 520 °C; (**c**) 530 °C; (**d**) 540 °C.

The samples underwent SEM testing to gain a better understanding of the α phase’s variations, and the results are displayed in Figure 6. Figure 6 reveals that the microstructure is a typical basket microstructure, consisting of residual β phase and interwoven amellar α_s_. In addition, the figure’s upper right corner provides the morphology of the α_GB_. It is apparent α_GB_ has a deformation-free, straight structure. The microstructure was subjected to TEM testing in order to further quantify the size variations in α_s_. Figure 7, which illustrates the results, shows that the distribution is erratic and that the size of α_s_ changes significantly with aging temperatures. The α_s_’s grain size is computed. The size of the α_s_ grows as the aging temperature rises, as seen in Figure 5b. This is because the higher the aging temperature, the faster the atoms diffuse, and the faster the grain grows. Additionally, the XRD test results were examined to determine the mass fraction of the α-phase, the findings are displayed in Figure 5b. As the aging temperature rises, the mass fraction of the α-phase first rises, then falls, and reaches its maximum at 530 °C. Remarkably, in contrast to earlier research, the α grain size and mass fraction exhibit distinct temperature variations. For example, Chen and Liu et al. discovered that while the mass fraction of the α-phase showed a monotonic declining trend, the α grain size grew as the aging temperature increased [23,29]. This should have something to do with how temperature affects the α-phase’s nucleation.

### 3.2. Effect of Heat Treatment on Alloy Mechanical Properties

#### 3.2.1. Effect of the Solution Time on the Mechanical Properties of the TB18 Alloy

**Figure 8:**
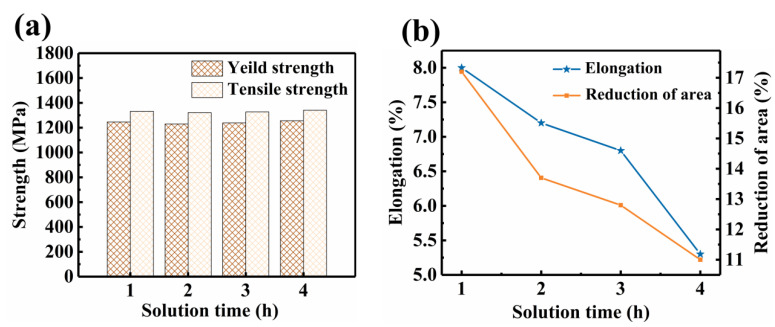
Tensile properties of TB18 alloy with same aging temperatures treatment after different solution treatment (870 °C/2 h). (**a**) Yield strength and tensile strength; (**b**) Elongation and Reduction of area.

To investigate the effect of the solution time on the properties of the alloys after aging, the alloy was tensile tested, and the results are shown in Figure 8. The selection of 530 °C as the aging temperature ensures the fastest precipitation of the α phase. After the aging treatment, the maximum change in yield strength (YS) and tensile strength was observed to be 26 MPa, indicating small effect on strength due to solution time, as shown in Figure 8a. Changes in strength are often associated with α phase. But as Figure 5 illustrates, the α-phase stays mostly constant as the solution time increases, leading to minimal intensity variation. However, as solution time increases, Figure 8b illustrates a declining tendency in both area reduction and elongation. The greatest changes in area reduction and elongation were 6.2% and 2.7%, respectively. It was noted that the trend of ductility change varied across different solution treatment time. During the period from 2 h to 3 h of solution treatment, the decrease in ductility was the slowest as the treatment time increased. Generally, the change in ductility is related to the α_GB_ and the prior β grain. The initiation and propagation of intergranular cracks preferentially occur along the continuous α_GB_, which is the primary cause of ductility degradation [30,31,32]. The smaller prior β-grain sizes make it more difficult for stress to concentrate and fracture to occur. In the samples subjected to 2 h of solution treatment, the α_GB_ is continuous, while in the samples treated for 3 h, the α_GB_ is discontinuous. However, the prior β-grain size in the latter is larger. As a result, the weakening effect of the discontinuous α_GB_ on ductility is offset by the larger prior β grains, leading to a slower decrease in ductility.

#### 3.2.2. Effect of Aging Temperature on the Mechanical Properties of the TB18 Alloy

**Figure 9:**
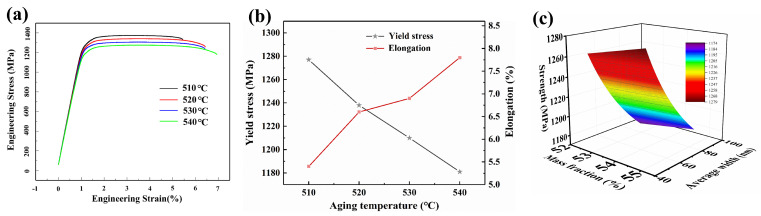
(**a**) The stress-strain curves of TB18 alloy with various aging temperatures. (**b**) The tensile properties of TB18 alloy at room temperature following treatment at various aging temperatures. (**c**) The fitted Hall-Petch relation.

Figure 9 displays the results of a tensile test conducted on the alloy to examine the impact of the aging temperature on the TB18 alloys’ properties. As the aging temperature rises, ductility increases and strength decreases, as seen in Figure 9a. The size and mass fraction of the α-phase are related to these variations in ductility and strength. The relationship between strength and α phase is explained by the Hall-Petch formula [33], in Equation (3),

(3) $$\sigma_{i}=C+\frac{K_{i}^{2}f_{\alpha }}{\sqrt{T_{\alpha }}}$$

where *σ_i_* represents the yield (*i* = *Y*) or tensile (*i* = *T*) strength, *K_i_*^2^ is a constant, *f*_α_ represent the volume fraction of α phases, *T*_α_ is the α thickness, and *C* represents a constant. By fitting the Hall-Petch formula, Equation (4) is obtained:

(4) $$\sigma_{i}=940.12+41.42\frac{f_{\alpha }}{\sqrt{T_{\alpha }}}$$

Figure 9b displays the fitted image. The *R*-value that fits is 0.925. According to the Hall-Petch relation, the strength rises as the mass fraction of the α-phase grows and falls as the α grain size increases. Grain boundaries, which prevent dislocations from moving, are more prevalent in finer α grains, increasing strength and decreasing ductility. The α-phase’s unequal size distribution could be the cause of the low goodness-of-fit.

## 4. Discussion

### 4.1. Effect of β Grain Refinement on Stress Distribution During Tensile Process

**Figure 10:**
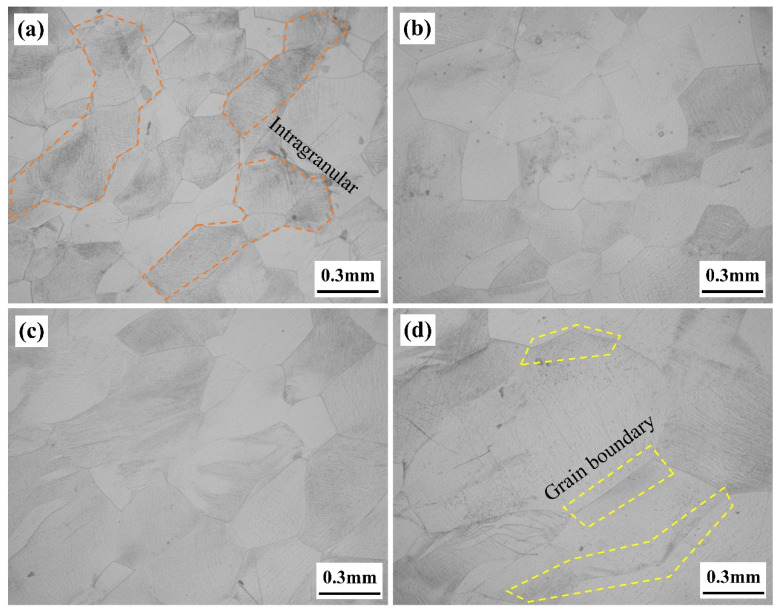
SEM images near the fracture of samples treated with different solution times. (**a**) 1 h; (**b**) 2 h; (**c**) 3 h; (**d**) 4 h. The orange dashed line is the stress concentration in the grain, and the yellow dashed line is the stress concentration at the grain boundary position.

The main goal of the solid solution stage for alloys treated above the phase transition point is to regulate the mechanical properties of the alloy by varying the size of the β grains. Cracks typically nucleate and propagate along the grain boundaries. There are more β grain boundaries the larger and more continuous they are, which causes stress to concentrate at these locations and increases the alloy’s susceptibility to fracture. SEM pictures of the fracture surfaces of tensile specimens with varying solution treatment times are displayed in Figure 10. By looking at the microstructure near the fracture, it can be observed that the distribution of tensile stress gets more heterogeneous as the β grain size grows. For the small β grains after a 1-h solution treatment, the stress is almost entirely distributed within the grains, as shown within the orange dashed line in Figure 10a. On the other hand, as seen by the yellow dashed line in Figure 10d, the stress near the grain boundaries increases whereas the stress within the bigger β grains reduces following a 4-h solution treatment. By allowing more grains to participate in the plastic deformation process, these observations imply that the substantial decrease in grain size effectively reduces stress concentration at the grain boundaries. This results in a higher degree of uniformity in the deformation, making breaks more difficult [34].

### 4.2. Effect of Aging Temperature on α Phase Precipitation

Given that the transformation of β phase to α phase in TB18 alloy is diffusion phase transition [35,36]. The precipitation process encompasses both nucleation and growth stages, which all influenced by the aging temperature [35]. At that time, when the aging began, α phase first began the nucleation process. The α-phase grew as the heating continued, and after adequate aging time, the mass fraction of the α-phase at the conclusion of the aging increased with the number of nucleations at the beginning. Atomic diffusion is a part of the α-phase’s growth process. Atomic mobility rises with temperature, which encourages the α-phase to expand. Uneven grain sizes result from the faster growth at higher temperatures, which causes α grains that have already nucleated to expand more quickly than those that have not.

The nucleation process is relatively more complex. Nucleation can be described by the nucleation rate. The nucleation rate is influenced by both the degree of supercooling and the diffusion ability of the atoms. When the aging temperature increases, the degree of supercooling decreases, the driving force of the nucleation decreases, but the atomic diffusion capacity increases. Therefore, at a certain aging temperature, the nucleation rate of the α phase reaches the maximum.

**Figure 11:**
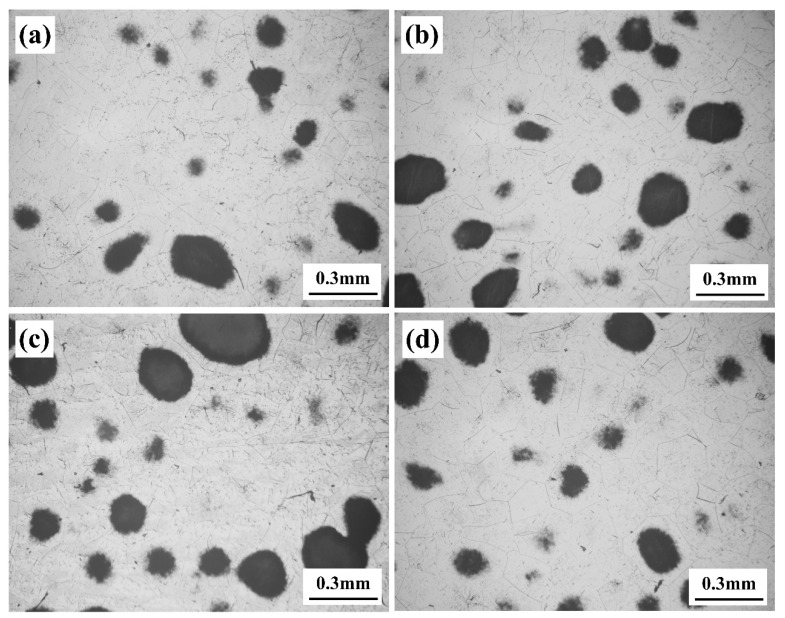
The metallographic images of samples were heat-treated for 5 min at different aging temperature. (**a**) 510 °C; (**b**) 520 °C; (**c**) 530 °C; (**d**) 540 °C.

**Figure 12:**
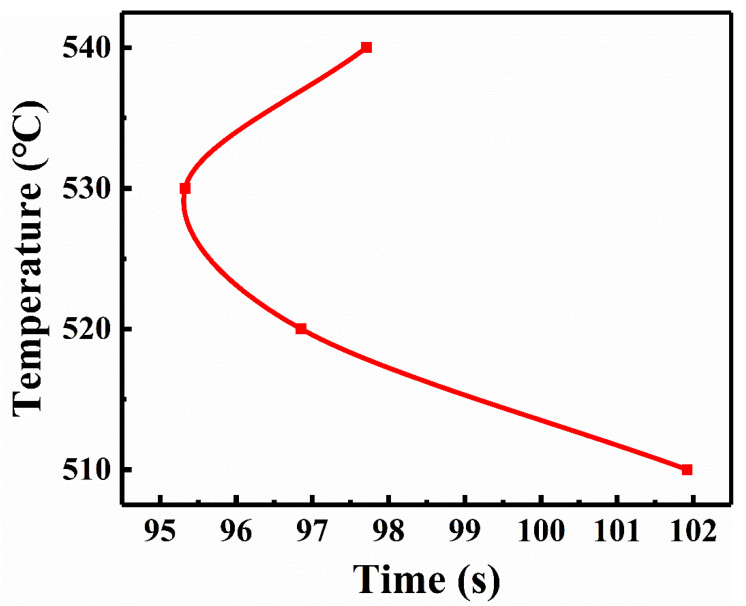
The time of gestation α phase at different aging temperatures by computational simulation.

The metallographic diagram of the samples at various aging temperatures after five minutes of age is displayed in Figure 11. The black part in the figure is the α phase of the initiation nucleus. The α phase has the most nucleation at 530 °C and forms most easily, so that the mass fraction of the α phase is maximized at this temperature. This is because the diffusion ability of the atoms has a main effect on the nucleation below 530 °C, thus the nucleation rate of the α phase increases as the aging temperature rises. However, above 530 °C, the degree of under cooling has a greater impact on the nucleation, so the aging temperature is further increased, and the nucleation rate of α phase decreases. Furthermore, the JMatPro program was used to simulate the gestation time of the α phase at different aging temperatures, producing Temperature Time Transformation (TTT) curves. The outcomes are displayed in Figure 12. The gestation time of the α phase aging at 530 °C is found to be the shortest, as shown in Figure 12. This indicates that the nucleation is fastest and nucleation rate is at its maximum at this temperature. This result suggests consistency between the experimental and theoretical predictions.

## 5. Conclusions

After examining the impact of aging temperature and solution duration on the mechanical characteristics and microstructure of TB18 alloy, the following results were found:

1. Longer solution times during the solution treatment cause the alloy’s β grain size to rise, which reduces the total β grain boundary. Additionally, stress concentration during the tensile process was successfully decreased by refining the β-phase grain size.
2. Throughout the aging treatment, the width of the α grain increases with increasing aging temperature. It is noteworthy that at 530 °C, the α phase precipitates the most, indicating its heightened sensitivity to this temperature. This sensitivity is attributed to the combined effect of temperature on supercooling.
3. Tensile tests on the alloy revealed that, for samples treated with the same aging temperature but with varying solution treatments, the alloy’s strength showed minimal change as the solution time increased. However, ductility steadily declined, and it was discovered that this decline was related to the size of the previous β grain.
4. After the same solution treatment, the TB18 alloy treated with varying aging temperatures shows a gain in strength but a decrease in ductility as the aging temperature rises. The Hall-Petch formula can be used to explain the correlation between these variations in strength and ductility and the size and mass fraction of the α grain.

In conclusion, the research presented in this paper provides a detailed analysis of the changes in the microstructure and mechanical properties of TB18 titanium alloy during the heat treatment process. The findings offer valuable insights for the future optimization of TB18 alloy properties and provide guidance for the control of its performance. Moreover, this study also serves as a useful reference for the heat treatment research of other near-β titanium alloys.

## Figures and Tables

**Table 1:** Heat treatment schemes of TB18 alloy.

Heat Treatment Scheme	Solution	Aging
HT1	870 °C/1 h/AC	530 °C/4 h/AC
	870 °C/2 h/AC	
	870 °C/3 h/AC	
	870 °C/4 h/AC	
HT2	870 °C/2 h/AC	510 °C/4 h/AC
		520 °C/4 h/AC
		530 °C/4 h/AC
		540 °C/4 h/AC

## Data Availability

The authors confirm that the data supporting the findings of this study are available within the article.
